# Psychosocial Adaptation After Heart Transplantation: The Chain-Mediating Effect of Self-Esteem and Death Anxiety on Social Support and Quality of Life in China

**DOI:** 10.3390/bs15101297

**Published:** 2025-09-23

**Authors:** Chan Gao, Song Gui, Lijun Zhu, Xiaoqian Bian, Heyong Shen, Can Jiao

**Affiliations:** 1 School of Psychology, Shenzhen University, Shenzhen 518060, China; gaochan@szu.edu.cn (C.G.); 2200482037@email.szu.edu.cn (S.G.); 2050042007@email.szu.edu.cn (L.Z.); 2 Institute of Analytical Psychology, City University of Macau, Macau 999078, China; h23092100231@cityu.edu.mo (X.B.); shenheyong@hotmail.com (H.S.); 3 School of Government, Shenzhen University, Shenzhen 518060, China; 4 The Shenzhen Humanities & Social Sciences Key Research Bases, Center for Mental Health, Shenzhen University, Shenzhen 518060, China

**Keywords:** heart transplantation, quality of life, social support, self-esteem, death anxiety, chain-mediation, Chinese culture

## Abstract

Heart transplantation represents a pivotal intervention for end-stage heart failure, extending survival. However, it imposes profound physical, psychological, and social challenges that often undermine recipients’ quality of life (QoL). These challenges are especially pronounced in collectivist cultural contexts like China, where familial obligations and stigma surrounding chronic illness intensify existential burdens. Grounded in theoretical frameworks including Coping Theory, Self-Determination Theory, Socioemotional Selectivity Theory, and Terror Management Theory, this cross-sectional study explored the interplay between social support and QoL among Chinese heart transplant recipients, elucidating the mediating roles of self-esteem and death anxiety, as well as their sequential chain-mediating pathway. Employing validated psychometric instruments, including the Social Support Rating Scale (SSRS), Rosenberg Self-Esteem Scale (RSES), Templer Death Anxiety Scale (T-DAS) and SF-36 Health Survey, along with chain-mediation modeling, the analysis revealed that social support exerts a direct positive influence on QoL, supplemented by indirect effects through enhanced self-esteem, reduced death anxiety, and a chained cognitive-existential mechanism linking these factors. These insights highlight the complex psychosocial dynamics of post-transplant adaptation, advocating for targeted and culturally attuned interventions. These interventions include family-based support programs, self-esteem enhancement strategies, and death anxiety counseling. The aim is to promote holistic rehabilitation and sustained well-being among heart transplant recipients in China’s context.

## 1. Introduction

Heart transplantation is a definitive treatment for end-stage heart failure, offering patients the opportunity to extend their lives. However, it also presents significant challenges in terms of physical, psychological, and social well-being (Levine et al., 2021). The quality of life (QoL) of heart transplant recipients is a critical indicator of transplant success (Dew et al., 2016; Religioni et al., 2025). According to the World Health Organization, QoL is defined as an individual’s perception of their position in life in relation to physical health, psychological well-being, social relationships, and environmental adaptation (The WHOQOL Group, 1995). Heart transplant recipients constitute a distinct patient population facing profound and persistent challenges in their post-transplant QoL (El Hadi et al., 2025; Fatma et al., 2021; Milaniak et al., 2020; Papalois & Papalois, 2023). In addition to managing complex medical regimens, including lifelong immunosuppressive therapy and the risks of organ rejection, these individuals often face significant psychological burdens such as death anxiety, identity disruption, guilt associated with organ donation, and difficulties in social reintegration (Mauthner et al., 2015; Papalois & Papalois, 2023; Shildrick, 2015; Svenaeus, 2012). In China, where collectivist values and familial obligations hold considerable importance, the societal stigma surrounding chronic illness and dependency may further exacerbate these challenges (Q. Ji et al., 2024; Schabert et al., 2013). According to China Cardiovascular Health and Disease Report 2022, there are approximately 6 million individuals with end-stage heart failure in China (China Cardiovascular Health and Disease Report Writing Group, 2023), for whom heart transplantation serves as the only viable treatment option. In 2024 alone, 1062 heart transplant procedures were performed nationwide, and this number continues to rise (Zheng, 2025). Despite the increasing number of transplants, research on the post-transplant QoL of recipients remains relatively underexplored in non-Western contexts. Chronic deficits in QoL are linked to adverse outcomes, including poor treatment adherence, psychological morbidity, and reduced long-term survival (Dew et al., 2016; Religioni et al., 2025). Therefore, in order to enhance the holistic recovery and well-being of this vulnerable population, it is imperative to investigate the determinants and mechanisms underlying their QoL experience.

### 1.1. Social Support and Quality of Life

Social support is an important determinant of psychological adaptation and is closely related to QoL. Social support refers to the help, care, and assistance that individuals receive within their social networks, including emotional, informational, and practical support (Cohen & Wills, 1985; Xiao, 1994). According to Lazarus and Folkman’s Coping Theory (Lazarus & Folkman, 1984), social support can alleviate the psychological burden individuals face when encountering stress and challenges, thereby enhancing their coping abilities and adaptability. The lack of social support, or low-quality social support, can lead to various negative psychological issues, such as anxiety, depression, and reduced life satisfaction (Cohen & Wills, 1985). Recent studies have indicated a positive relationship between social support and QoL (Jiang et al., 2025; Kristianto & Yudiarso, 2025). Individuals who receive higher levels of social support are more likely to experience greater life satisfaction, more stable psychological states, and a reduction in the distress and challenges they face in daily life (Ban et al., 2021; Ding et al., 2024; D. Zhang, 2021). Research also demonstrates that social support significantly enhances QoL among individuals facing major health conditions or life changes (Adejumo et al., 2025; Dun et al., 2022; Thompson et al., 2022). Longitudinal studies have further confirmed that social support exerts a significant impact on an individual’s long-term QoL (Cho et al., 2024). This effect has also been replicated in empirical research among other chronic illness populations, indicating that enhancing levels of social support contributes to improved patient QoL (D. Lin et al., 2024; Luo et al., 2023). Although extensive research has consistently affirmed the positive association between social support and QoL, the specific mechanisms underlying this relationship in heart transplant recipients remain unclear and warrant further investigation in future studies.

### 1.2. The Mediating Role of Self-Esteem

Self-esteem is a fundamental component of psychological well-being. It involves an individual’s global appraisal of their own worth, competence, and lovability across diverse life domains (Rosenberg, 1965). Research has shown a strong positive association between self-esteem and QoL. Individuals with higher self-esteem tend to adopt health-promoting behaviors, maintain effective coping strategies, and pursue meaningful personal and social goals, ultimately resulting in a higher overall QoL (Barbalat et al., 2022; Sadjapong & Thongtip, 2023). Self-Determination Theory suggests that self-esteem is fostered through the satisfaction of basic psychological needs, which in turn contributes to greater psychological well-being and overall QoL (Deci & Ryan, 2000). Individuals with high levels of self-esteem tend to view themselves as capable and worthy, which fosters a more positive outlook when facing challenges (Reed et al., 2021). They are more likely to engage in proactive coping, maintain optimism, and pursue meaningful goals, ultimately contributing to a higher QoL (Chung et al., 2021).

In addition, self-esteem is influenced by social support. Individuals who receive lower levels of emotional or instrumental support from their social environment are more likely to develop lower self-esteem, as they may interpret the lack of support as a reflection of low personal worth or inadequacy (Goodwin et al., 2004; S. Lin et al., 2022; Surzykiewicz et al., 2022). Social support also shapes how individuals evaluate themselves in relation to others. Those with insufficient social support may internalize negative feedback or experience greater self-doubt in social contexts, which can erode their sense of competence and self-worth over time (van Schie et al., 2018). Given that social support influences self-esteem and self-esteem affects QoL, self-esteem likely mediates this relationship.

### 1.3. The Mediating Role of Death Anxiety

Death anxiety refers to individuals’ fear, apprehension, or worry about death and the process of dying (Lehto & Stein, 2009). It encompasses existential concerns about the loss of self, uncertainty about the afterlife, and anxiety about the potential suffering associated with death (Iverach et al., 2014). According to the Terror Management Theory, death anxiety can significantly influence individuals’ psychological and behavioral functioning, especially when their physical vulnerability is heightened (Greenberg et al., 1986; Svet et al., 2023). Research indicates that death anxiety can negatively affect individuals’ overall QoL, particularly among those coping with chronic or life-threatening illnesses (Hong et al., 2022). Furthermore, individuals who constantly worry about death may experience difficulties engaging in meaningful activities, maintaining hope for the future, or cultivating a positive outlook, thereby reducing their QoL (Merati et al., 2024). In the context of heart transplantation, patients may face ongoing concerns about graft failure, complications, or mortality, which can further intensify death-related fears and limit their psychological recovery.

Moreover, death anxiety demonstrates a strong inverse association with social support. According to research, individuals who report higher levels of emotional, informational, or instrumental support from their family, friends, or healthcare providers tend to experience significantly lower levels of death-related fear and existential distress (Hajihasani & Naderi, 2021; Khanbabai Ghaleie et al., 2024). In contrast, individuals with limited access to reliable support networks often face greater uncertainty and helplessness when confronting issues related to mortality, thereby intensifying their death anxiety (Huang et al., 2022). In medical populations such as heart transplant recipients, consistent and compassionate support from caregivers not only provides reassurance but also reinforces patients’ sense of safety and belonging, which can buffer the psychological impact of life-threatening illness (Grossman, 2022). Furthermore, studies have shown that social support is negatively correlated with anxiety and depressive symptoms, both of which are closely linked to elevated death anxiety (Kandeğer et al., 2021). Thus, death anxiety may mediate by amplifying the negative effects of low social support on QoL.

### 1.4. The Chain-Mediating Role of Self-Esteem and Death Anxiety

There is a strong correlation between self-esteem and death anxiety. Studies have demonstrated that individuals with low self-esteem are more likely to experience heightened fear of death, as a fragile sense of self may intensify feelings of vulnerability and existential insecurity (Guan et al., 2020; Huang et al., 2022). According to the Socioemotional Selectivity Theory, when individuals perceive time as limited—such as in the case of serious illness—they tend to prioritize emotionally meaningful goals, including the maintenance of self-worth and close interpersonal bonds (Carstensen, 2006). Individuals with low self-esteem frequently experience heightened perceptions of insignificance and inadequacy when confronted with mortality (Smirnov & Makarova, 2025). Consequently, to manage existential distress and reduce psychological vulnerability, they may amplify death-related anxiety as they lack the psychological resources to buffer existential threats (Vail et al., 2020). While social support networks provide meaningful buffers against existential concerns, their perceived adequacy and stability directly modulate the salience of death anxiety, subsequently influencing how individuals process mortality awareness in daily functioning (Kisomi et al., 2024). Therefore, social support may affect QoL through the chain-mediating effect of self-esteem and death anxiety.

### 1.5. Cultural Context of Heart Transplantation in China

The psychosocial adaptation of heart transplant recipients in China is profoundly shaped by its collectivist cultural context. Values such as familial obligation, social harmony, and “face” (mianzi) create a complex dynamic for recovery (Kuo, 2013; Sun et al., 2023). While providing a strong support network, these values can also intensify stigma, where chronic illness is sometimes perceived as a personal failing or a burden to the family (Smith et al., 2017). Cultural narratives that attribute illness to lifestyle factors (e.g., diet, stress) may exacerbate feelings of shame, potentially inhibiting open support-seeking to avoid bringing shame to the family (Clair et al., 2016).

This is further influenced by Traditional Chinese Medicine (TCM) perspectives, where the heart (心, xīn) is regarded as the seat of spirit and emotions. Within the framework of TCM, a heart ailment is often conceptualized as a manifestation of qi deficiency or emotional imbalance, which profoundly influences patients’ illness perceptions and recovery expectations (L.-S. Wang et al., 2022). This holistic view underscores the cultural linkage between physical and psychosocial well-being. Therefore, the experiences of self-esteem and death anxiety are filtered through these cultural lenses, where threats to one’s social identity and symbolic meaning of the heart can uniquely intensify psychological distress. This study is situated within this framework, hypothesizing that these cultural nuances are captured in the mediating roles of self-esteem and death anxiety.

### 1.6. The Current Study

**Figure 1:**
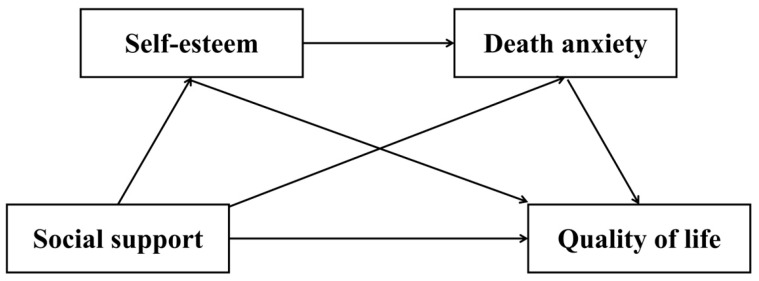
Hypothetical model of the study.

The post-transplantation period represents a critical phase wherein recipients navigate profound physiological challenges and seek to rebuild social connections to enhance their compromised QoL. While substantial research has established correlations between social support and QoL in clinical populations, few studies have investigated the underlying psychological mechanisms specific to heart transplant recipients, particularly within non-Western cultural contexts. To address this gap, the present study is grounded in an integrative theoretical framework that illuminates the psychosocial and existential dimensions of post-transplant adaptation. Drawing upon Coping Theory (Lazarus & Folkman, 1984), we position social support as a critical resource that buffers transplant-related stressors and enhances adaptive functioning. Furthermore, Self-Determination Theory (Deci & Ryan, 2000) informs how this support fulfills fundamental psychological needs for relatedness and competence, thereby fostering self-esteem—a key cognitive resource for positive identity integration. Conversely, Terror Management Theory (Greenberg et al., 1986) provides the existential lens through which we examine how social support mitigates death anxiety by reinforcing cultural worldviews and providing a sense of symbolic immortality, which is particularly salient in the context of a life-threatening condition and a new organ. This relationship is further contextualized by Socioemotional Selectivity Theory (Carstensen, 2006), which posits that a heightened perception of limited time, as experienced by transplant recipients, leads to a prioritization of emotionally meaningful goals and social bonds, making the quality of one’s self-worth and social connections paramount. Integrating these perspectives, this cross-sectional study examines the relationship between social support and QoL among Chinese heart transplant recipients, with a specific focus on the chain-mediating roles of self-esteem and death anxiety. We propose that social support not only directly enhances QoL but also operates through a sequential cognitive-existential pathway: by bolstering self-esteem, which in turn provides the psychological resources to reduce death anxiety, ultimately leading to improved QoL. This research aims to elucidate these mechanisms within China’s collectivist cultural context, where familial interdependence and symbolic meanings attached to the heart may uniquely shape these pathways, thereby providing critical insights for enhancing psychosocial rehabilitation. The study hypothesizes that (Figure 1):

**Hypothesis 1.**  *Social support positively predicts QoL among heart transplant recipients.*

**Hypothesis 2.**  *Self-esteem mediates the relationship between social support and QoL.*

**Hypothesis 3.**  *Death anxiety mediates the relationship between social support and QoL.*

**Hypothesis 4.**  *Self-esteem and death anxiety sequentially mediate the social support-QoL relationship (social support → self-esteem → death anxiety → QoL).*

## 2. Materials and Methods

### 2.1. Participants

A total of 420 heart transplant recipients were initially recruited for the study. Five participants were excluded from the final analysis due to incomplete responses on the survey questionnaires. Thus, 415 participants were included for the analyses, resulting in a completion rate of 98.81%. Among the participants, there were 273 (65.78%) males and 142 (34.22%) females; 220 (53.01%) were aged 19–44, 156 (37.59%) were 45–59, and 39 (9.40%) were 60–66 years old. In terms of education background, 129 (31.08%) had high school or vocational education, 146 (35.18%) held an associate degree, 105 (25.30%) had an undergraduate degree, and 35 (8.43%) had a master’s degree or higher. Regarding heart transplant duration, 92 (22.17%) had undergone transplantation for less than a year, 198 (47.71%) for 1–5 years, 106 (25.54%) for 6–10 years, and 19 (4.58%) for 11–17 years. In addition, 286 (68.92%) participants resided in urban areas and 129 (31.08%) in rural areas.

### 2.2. Measures

#### 2.2.1. Social Support Rating Scale

The Social Support Rating Scale (SSRS) developed by Xiao (1994) was used in this study, with the Chinese version of the scale. The scale consists of 10 items (e.g., “How many close friends do you have who can provide support and help?”), divided into three dimensions: subjective support (items 1, 3, 4, 5), objective support (items 2, 6, 7), and support utilization (items 8, 9, 10). Items are scored on a 4-point Likert scale (1 = none to 4 = full support). Higher scores indicate higher levels of social support. The total score ranges from 12 to 66, with scores below 22 indicating low support, 23–44 general support, and 45–66 satisfactory support. In this study, the Cronbach’s alpha coefficient for this scale was found to be 0.888.

#### 2.2.2. Rosenberg Self-Esteem Scale

The Rosenberg Self-Esteem Scale (RSES) was employed to measure self-esteem, using the Chinese version (P. Wang et al., 1998). The scale comprises 10 items (e.g., “I feel that I am a person of worth, at least on an equal plane with others”), with 4 items reverse-scored (items 3, 5, 9, 10). It utilizes a 4-point Likert scale (1 = strongly disagree to 4 = strongly agree). A higher total score corresponds to higher self-esteem. In this study, the Cronbach’s alpha coefficient for this scale was found to be 0.855.

#### 2.2.3. Templer Death Anxiety Scale

This study employs the Chinese version of the Templer Death Anxiety Scale (T-DAS) revised by Yang et al. (2013), which has demonstrated good reliability and validity in older adults. The scale consists of 15 items (e.g., “I am very afraid of death”), encompassing four dimensions: emotion, stress and distress, time, and cognition, with 6 items reverse-scored (items 2, 3, 5, 6, 7, 15). It uses a 5-point Likert scale (1 = strongly disagree to 5 = strongly agree). A higher score indicates greater death anxiety. In this study, the Cronbach’s alpha coefficient for this scale was found to be 0.885.

#### 2.2.4. SF-36 Health Survey

The SF-36 Health Survey, developed by the Boston Health Research Institute, was used to assess QoL, employing the Chinese version (Ware et al., 1993). The scale includes 36 items across eight dimensions: physical functioning (PF), role-physical (RP), bodily pain (BP), general health (GH), vitality (VT), social functioning (SF), role-emotional (RE), and mental health (MH), grouped into physical health (PCS) and mental health (MCS) components. Scoring involves a standardized transformation: converted score = (actual score—minimum possible score)/(maximum possible score—minimum possible score) × 100, with higher scores indicating better QoL. The total score is the average of the eight dimensions, PCS is the average of PF/RP/BP/GH, and MCS is the average of VT/SF/RE/MH. In this study, the Cronbach’s alpha coefficient for this scale was found to be 0.915.

### 2.3. Procedures

This study was conducted across three high-volume cardiac transplant centers located in northern, central, and southern provinces of China. Eligible participants were identified by clinical staff from hospital registries based on the following inclusion criteria: (1) adults aged ≥ 18 years; (2) survival > 1-month post-transplant with successful hospital discharge; (3) intact cognitive function and communication capacity; (4) ability to provide voluntary informed consent. Exclusion criteria included: (1) cognitive impairment (assessed via a mental status examination score < 24); (2) severe comorbidities; (3) communication barriers (e.g., aphasia, hearing/visual impairments).

The survey was administered electronically via the Wenjuanxing platform (www.wjx.cn), a secure and certified online questionnaire tool widely used in China. The platform’s certified authentication process ensured that consent was obtained digitally before participants could access the survey. The questionnaire took approximately 15–20 min to complete. Participants had the right to withdraw at any time without affecting their medical care.

Prior to the survey, institutional ethics approval was obtained from the Ethics Committee of Medical School, Shenzhen University (Approval No.: PN-202500115). All procedures used in this study were conducted in accordance with general ethical guidelines in psychology and adhered to the principles of the Declaration of Helsinki.

All responses were anonymized through unique participant codes, with personal identifiers permanently excluded. Real-time data encryption was implemented during transmission, and encrypted datasets were securely stored on password-protected servers. Physical copies of data were permanently destroyed post-analysis. Confidentiality protocols were rigorously enforced throughout the study.

### 2.4. Data Analysis

Data analysis was performed using SPSS Statistics (Version 27.0; IBM Corp., 2023). First, descriptive statistics (means, standard deviations, frequencies) were computed for all study variables. Second, Harman’s single-factor test was conducted to assess common method variance. Third, Pearson correlation analyses were performed to examine the bivariate relationships between the key variables.

The primary analysis tested the hypothesized chain-mediation model using Hayes’ PROCESS macro (Version 4.2, Model 6) (Hayes, 2017). The significance of the direct and indirect effects was determined using bootstrap confidence intervals based on 5000 bootstrap samples. A 95% confidence interval that did not include zero indicated a statistically significant effect.

## 3. Results

### 3.1. Common Method Variance Test

Given that the data in this study were collected via self-report measures from participants, common method variance (CMV) could potentially be present. To evaluate the extent of common method bias, Harman’s single-factor test was performed (Xiong et al., 2012). The analysis revealed 15 factors with eigenvalues exceeding 1, with the first factor accounting for 20.992% of the variance—well below the conventional threshold of 40% (Podsakoff et al., 2003). Consequently, no significant common method variance was evident in the present study.

### 3.2. Descriptive Statistics and Correlation Analysis

Pearson correlation analyses were performed on all variables, with descriptive statistics and correlation results displayed in Table 1. Findings indicated that social support was significantly positively correlated with self-esteem and QoL, while showing a significant negative correlation with death anxiety. Self-esteem demonstrated a significant positive correlation with QoL and a significant negative correlation with death anxiety. Furthermore, death anxiety exhibited a significant negative correlation with QoL. Given that gender was significantly correlated with death anxiety and QoL, age showed significant correlations with death anxiety and QoL, and Heart transplantation years positively correlated with QoL, these three variables were included as covariates in the subsequent analyses.

### 3.3. Mediation Effect Analysis

**Figure 2:**
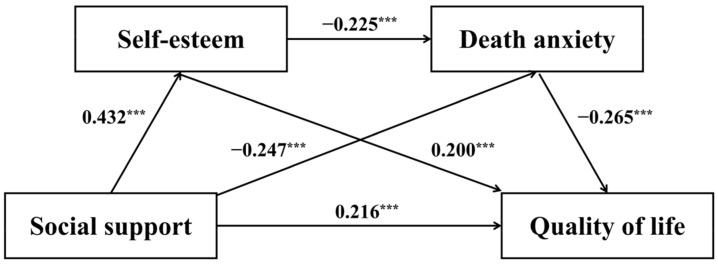
Chain-mediating effect of self-esteem and death anxiety (*** *p* < 0.001).

In the present study, Hayes’ SPSS (IBM Corp., 2023) macro PROCESS v4.2 (Model 6) was employed to examine the chain-mediation effects (Hayes, 2017), utilizing 5000 bootstrap samples and 95% confidence intervals, while controlling for participants’ gender, age, and heart transplantation years. Regression analysis results revealed that social support had a significant positive predictive effect on QoL (*β* = 0.216, *t* = 4.602, *p* < 0.001) and self-esteem (*β* = 0.432, *t* = 9.653, *p* < 0.001), alongside a significant negative predictive effect on death anxiety (*β* = −0.247, *t* = −4.982, *p* < 0.001). Furthermore, self-esteem demonstrated a significant negative predictive effect on death anxiety (*β* = −0.225, *t* = −4.562, *p* < 0.001) and a positive predictive effect on QoL (*β* = 0.200, *t* = 4.297, *p* < 0.001). Additionally, death anxiety significantly and negatively predicted QoL (*β* = −0.265, *t* = −5.826, *p* < 0.001). Detailed results are detailed in Table 2 and Figure 2.

As illustrated in Table 3, additional mediation analysis indicated that self-esteem and death anxiety served as significant mediators in the association between social support and QoL. The total mediating effect represented 45.1% of the overall effect and comprised three distinct pathways. The first pathway involved the independent mediating role of self-esteem (mediating effect = 0.086, SE = 0.021, bootstrap 95% CI: [0.046, 0.130]), accounting for 21.9% of the total effect. The second pathway consisted of the independent mediating role of death anxiety (mediating effect = 0.065, SE = 0.018, bootstrap 95% CI: [0.034, 0.103]), which accounted for 16.6% of the total effect. The third pathway was the chain-mediating effect through self-esteem and death anxiety (mediating effect = 0.026, SE = 0.007, bootstrap 95% CI: [0.013, 0.042]), representing 6.6% of the total effect. Thus, the findings demonstrated that social support not only directly predicted QoL but also exerted indirect influences via the independent mediating effects of self-esteem and death anxiety, as well as through the sequential chain from self-esteem to death anxiety, thereby supporting the proposed hypothesis.

## 4. Discussion

This study examined the relationship between social support and QoL among Chinese heart transplant recipients, focusing on the mediating roles of self-esteem and death anxiety within a collectivist cultural framework. Our findings confirmed all hypotheses: social support positively predicted QoL, with self-esteem and death anxiety acting as individual mediators, and their sequential mediation partially explaining the pathway (social support → self-esteem → death anxiety → QoL), underscoring the cognitive-existential mechanisms that underpin post-transplant psychosocial adjustment. The total indirect effect accounted for 45.1% of the total effect, highlighting the substantial role of these mediators.

### 4.1. Effect of Social Support on Quality of Life

Our findings confirm Hypothesis 1, demonstrating that social support significantly and positively predicts QoL in Chinese heart transplant recipients, even after controlling for gender, age, and heart transplantation years. Grounded in Lazarus and Folkman’s Coping Theory (Lazarus & Folkman, 1984), which posits that social resources buffer stress by enhancing adaptive coping, this direct pathway underscores how emotional and instrumental support from family and peers mitigates transplant-specific stressors, such as chronic immunosuppression, organ rejection anxiety, and identity disruption post-surgery (El Hadi et al., 2025; Hu et al., 2024; Y. Wang et al., 2025). Extending this perspective, we further emphasize that not all forms of support are equally impactful. Drawing on Zech et al. (2004), the quality of support, particularly the presence of empathetic listening and emotional validation, may matter more than the quantity of social interactions, especially when confronting profound existential and identity-related distress. Consistent with prior longitudinal studies, robust social networks are associated with improved emotional well-being and reduced isolation in long-term heart transplant survivors (Ross et al., 2010; Yan et al., 2022). However, our results diverge from research in broader cardiovascular cohorts, where social integration primarily promotes treatment adherence and lowers morbidity risks (Rosenberger et al., 2012), by showing a stronger existential focus—such as grappling with donor-related guilt and mortality salience—rather than just adherence promotion. These challenges are particularly in Chinese culture, emphasizing the role of social support in fostering holistic psychosocial recovery beyond symptom management.

This cultural context further elucidates the findings: familial interdependence, a hallmark of Chinese society, transforms social support into a protective mechanism against stigma and dependency burdens, thereby enhancing QoL domains like physical vitality and social reintegration (Gao et al., 2025). Within this collectivist framework, however, emotional sharing may be implicitly discouraged to avoid familial burden, potentially limiting opportunities for deep emotional processing (Rimé et al., 2020). Thus, while instrumental and informational support remain salient, the absence of high-quality emotional support may constrain psychosocial recovery. Unlike individualistic cultures, where self-reliance might dilute social support’s impact (Ishikawa et al., 2023), our study uniquely quantifies how collectivist values amplify this pathway, addressing a gap in non-Western transplant research and emphasizing social support’s role in fostering holistic psychosocial adjustment beyond symptom management.

### 4.2. The Mediating Role of Self-Esteem and Death Anxiety

Building on the direct effects of social support, our results support Hypothesis 2 by confirming self-esteem as a mediator in the social support-QoL relationship among heart transplant recipients, with an independent mediation effect of 21.9%. Drawing from Self-Determination Theory (Deci & Ryan, 2000), which emphasizes that the fulfillment of psychological needs like competence and relatedness fosters self-worth, social support enhances self-esteem by providing validation and emotional security, thereby enabling better coping with post-transplant identity shifts and bodily integration. This mediation aligns with recent chronic disease studies, where perceived support indirectly boosts QoL through self-esteem-building resilience (P. Ji et al., 2024; C. Lin et al., 2025). Our findings in the heart transplant context extend general self-management models, such as those for coronary heart disease where self-esteem mainly affects emotional domains (Arsyi et al., 2022), by emphasizing self-esteem’s critical role in addressing heart transplantation’s unique existential challenges. These include integrating a ‘foreign’ organ, navigating donor-related guilt, and managing heightened death anxiety, particularly amplified in China’s collectivist culture where the heart holds profound symbolic significance as the core of life and moral identity (Zhou et al., 2023).

In China’s collectivist framework, this mediation is amplified, as familial support reinforces self-worth through interdependent validation, differing from individualistic settings where self-reliance may predominate (X. Liu & Bai, 2024). Critically, our analysis improves a limitation in prior liver transplant research, which focuses on self-esteem as a distress buffer without accounting for heart-specific uncertainties like graft failure (D. Zhang et al., 2023); here, self-esteem’s mediating strength in our data underscores its necessity for physical adherence and emotional reintegration, offering a novel cultural adaptation to existing models (Wu et al., 2024).

Furthermore, Hypothesis 3 is validated, with death anxiety mediating the social support-QoL link, contributing an independent mediation effect of 16.6%. Informed by Terror Management Theory (Greenberg et al., 1986), which asserts that social buffers mitigate mortality fears by affirming cultural worldviews, our results indicate that social support reduces death anxiety through the provision of subjective support, objective support, and support utilization, ultimately leading to an improved quality of life. This pathway echoes findings in hemodialysis and cardiovascular disorder patients, where weak support exacerbates existential distress and erodes daily engagement (Hashim et al., 2022; Jaberi et al., 2025). However, heart transplantation introduces amplified mortality salience due to graft complications, cultural taboos in China such as organ donation stigma, and the profound cultural significance of the heart, leading Chinese heart transplant recipients to face greater death anxiety compared to other illness groups (Z. Zhang et al., 2022). Our study confirms the impact of death anxiety on QoL in Chinese heart transplant recipients and, by quantifying its mediating role in the social support-QoL relationship, demonstrates that social support’s anxiety-alleviating effects are more potent in collectivist contexts, where familial buffers counteract isolation-driven fears, thus providing a culturally tailored mechanism (J. Liu et al., 2024).

### 4.3. The Chain Mediation Through Self-Esteem and Death Anxiety

Our findings further substantiate Hypothesis 4, demonstrating a sequential mediation pathway where social support influences self-esteem, which in turn affects death anxiety, ultimately impacting QoL, with a chain-mediation effect of 6.6%. By integrating Coping Theory and Self-Determination Theory for cognitive adaptation with Socioemotional Selectivity Theory and Terror Management Theory for existential buffering, this study constructs a chained cognitive-existential mechanism, as posited in the hypotheses, where social support enhances self-esteem to mitigate death anxiety and ultimately improve QoL. This pathway elucidates the cognitive-existential mechanisms underpinning post-transplant adjustment. Social support first bolsters self-esteem by satisfying needs for relatedness and competence, as per Self-Determination Theory (Deci & Ryan, 2000). This, in turn, diminishes death anxiety by fostering existential stability and reducing vulnerability to mortality threats. This effect is particularly evident when perceived time limitations prioritize emotional goals, according to Socioemotional Selectivity Theory (Carstensen, 2006), and when cultural worldviews buffer mortality fears, per Terror Management Theory (Greenberg et al., 1986). Ultimately, these processes enhance QoL. This chain aligns with chronic illness research, where social support indirectly improves health through self-esteem and reduced depressive symptoms (G. Li et al., 2023; Z. Li et al., 2025). However, our model extends previous mediation approaches in heart transplant research, such as those examining the partial mediation effect of satisfaction with social support and coping effectiveness on health-related QoL (White-Williams et al., 2014), by emphasizing the sequential role of self-esteem and death anxiety, particularly in addressing existential threats heightened by organ rejection and donor guilt.

In contrast to kidney transplant studies, which often prioritize coping strategies in the support-QoL linkage (Knobbe et al., 2025), our findings in heart transplantation emphasize self-esteem as a foundational precursor in the chain mediation process. This chain is particularly relevant here due to the unique aspects of heart transplantation, such as the symbolic significance of the heart in Chinese culture—representing the core of emotion, life essence, and moral identity—which intensifies existential challenges like profound identity disruption, donor guilt, and heightened death anxiety. Consequently, the sequential pathway more effectively captures these amplified cognitive-existential dynamics, where social support bolsters self-worth to directly mitigate mortality fears, especially in a collectivist context that amplifies familial interdependence and cultural-specific anxieties like familial burden (Zhou et al., 2023). This integrated chain model captures the interplay of cognitive and existential factors, advancing theoretical frameworks beyond isolated mediators. Moreover, it offers a robust foundation for designing interventions that target both self-esteem and death anxiety to optimize post-transplant QoL.

## 5. Implications

The post-transplant phase demands addressing both physiological and psychosocial needs to optimize recipients’ well-being. This cross-sectional analysis elucidates how social support influences QoL via self-esteem and death anxiety in China’s cultural context, providing a foundation for targeted strategies. Results indicate that recipients with strong support experience bolstered self-esteem, mitigating death anxiety and isolation, which aligns with interventions improving mental health in cardiovascular populations (Zambrano et al., 2020). Theoretically, it advances models of psychosocial recovery by highlighting chain mechanisms, informing frameworks for chronic illness management. Practically, interventions should include family-based support programs to enhance self-esteem through validation, alongside death education to reduce anxiety in waiting and post-transplant phases. In China, culturally sensitive approaches like community counseling could address stigma, potentially lowering morbidity and healthcare burdens (Qin et al., 2024). Broader applications extend to policy, advocating for integrated psychosocial screening in transplant protocols to optimize outcomes and donor–recipient dynamics. Future efforts might incorporate digital tools for real-time support, enhancing accessibility in rural areas and promoting long-term QoL.

## 6. Limitations and Research Perspectives

Despite its contributions, this study has limitations inherent to cross-sectional designs, which restrict causal inferences despite robust mediation paths. Longitudinal tracking could elucidate temporal dynamics in psychosocial trajectories (Hettwer et al., 2024). The sample’s urban Chinese focus may limit generalizability to rural or global populations, where cultural and resource variances influence psychosocial factors (Saltzman & Hansel, 2024). Self-reported data risks bias from recall or desirability, though validated scales help; incorporating objective measures like behavioral observations or biomarkers would strengthen validity (Fillingim et al., 2025). Sample size constraints might overlook subgroups, such as pediatric or multi-organ transplants. Future research should employ prospective designs with diverse cohorts to validate causality and explore moderators like multimorbidity. Qualitative integrations could capture nuanced experiences, while intervention trials testing support enhancements would address practical gaps. Cross-cultural comparisons might reveal global applicability, informing equitable transplant psychosocial care.

## 7. Conclusions

This study reveals that social support not only directly predicts QoL but also indirectly through self-esteem and death anxiety’s independent and chain mediations among heart transplant recipients. Chinese collectivist values such as family duty and harmony shape psychosocial mechanisms, making support abundant but often focused on practical aid, influenced by concerns about face and avoiding burden. This cultural nuance suggests that the quality and type of support, particularly the perceived availability of unconditional emotional support, may be as important as its quantity in fostering self-esteem and alleviating existential fears. By delineating these mechanisms in a Chinese context, it contributes novel insights to transplant psychology, advocating for multifaceted interventions to bolster psychosocial resilience and long-term QoL.

## Figures and Tables

**Table 1:** Descriptive statistics and correlation analysis of variables (*N* = 415).

Variables	*M*	*SD*	1	2	3	4	5	6	7
1 Gender	-	-	1						
2 Age	43.860	11.276	−0.009	1					
3 Heart transplantation years	4.380	3.326	0.087	0.048	1				
4 Social support	36.670	9.388	−0.089	−0.046	0.069	1			
5 Self-esteem	24.650	5.217	−0.015	−0.082	0.038	0.433 ***	1		
6 Death anxiety	46.580	10.444	0.097 *	0.142 **	−0.089	−0.362 ***	−0.345 ***	1	
7 Quality of life	48.410	17.291	−0.121 *	−0.112 *	0.160 **	0.417 ***	0.395 ***	−0.439 ***	1

Note. * *p* < 0.05, ** *p* < 0.01, *** *p* < 0.001; Gender: male = 1, female = 2.

**Table 2:** Multiple regression analysis (*N* = 415).

Regression Equation		Fitting Indicator		Coefficient Significance	
Outcome Variables	Predictor Variables	*R* ^2^	*F*	*β*	*t*
Self-esteem	Gender	0.192	24.407 ***	0.023	0.502
	Age			−0.062	−1.388
	Heart transplantation years			0.009	0.209
	Social support			0.432	9.653 ***
Death anxiety	Gender	0.198	20.158 ***	0.079	1.775
	Age			0.116	2.616 **
	Heart transplantation years			−0.076	−1.697
	Social support			−0.247	−4.982 ***
	Self-esteem			−0.225	−4.562 ***
Quality of life	Gender	0.322	32.220 ***	−0.084	−2.030 *
	Age			−0.055	−1.318
	Heart transplantation years			0.124	2.997 **
	Social support			0.216	4.602 ***
	Self-esteem			0.200	4.297 ***
	Death anxiety			−0.265	−5.826 ***

Note. * *p* < 0.05, ** *p* < 0.01, *** *p* < 0.001.

**Table 3:** Indirect effects of Self-esteem and Death anxiety.

Path	Effect Value	Boot SE	Boot LLCI	Boot ULCI	Relative Mediation Effect
Path1: SS → SE → QoL	0.086	0.021	0.046	0.130	21.9%
Path2: SS → DA → QoL	0.065	0.018	0.034	0.103	16.6%
Path3: SS → SE → DA → QoL	0.026	0.007	0.013	0.042	6.6%
Total indirect effect	0.178	0.026	0.129	0.231	45.1%

Note. SS = Social support, SE = Self-esteem, DA = Death anxiety, QoL = Quality of life; BootSE = Bootstrap standard error, Boot LLCI = Bootstrap lower limit confidence interval, Boot ULCI = Bootstrap upper limit confidence interval.

## Data Availability

The datasets used and/or analyzed during the current study are available from the corresponding author on reasonable request, with ethical approval from the institutional review board.

## Abbreviations

The following abbreviations are used in this manuscript:

QoL	Quality of Life
SS	Social Support
SE	Self-Esteem
DA	Death Anxiety

## References

[B1-behavsci-15-01297] Adejumo O. A., Jinabhai C., Daniel O., Haffejee F. (2025). The effects of stigma and social support on the health-related quality of life of people with drug resistance tuberculosis in Lagos, Nigeria. Quality of Life Research.

[B2-behavsci-15-01297] Arsyi D. H., Permana P. B. D., Karim R. I. (2022). The role of optimism in manifesting recovery outcomes after coronary artery bypass graft surgery: A systematic review. Journal of Psychosomatic Research.

[B3-behavsci-15-01297] Ban Y., Li M., Yu M., Wu H. (2021). The effect of fear of progression on quality of life among breast cancer patients: The mediating role of social support. Health and Quality of life Outcomes.

[B4-behavsci-15-01297] Barbalat G., Plasse J., Gauthier E., Verdoux H., Quiles C., Dubreucq J., Legros-Lafarge E., Jaafari N., Massoubre C., Guillard-Bouhet N. (2022). The central role of self-esteem in the quality of life of patients with mental disorders. Scientific Reports.

[B5-behavsci-15-01297] Carstensen L. L. (2006). The influence of a sense of time on human development. Science.

[B6-behavsci-15-01297] China Cardiovascular Health and Disease Report Writing Group (2023). Summary of china cardiovascular health and disease report 2022. Chinese Circulation Journal.

[B7-behavsci-15-01297] Cho H., Kang D., Shin D. W., Kim N., Lee S. K., Lee J. E., Nam S. J., Cho J. (2024). Social support during re-entry period and long-term quality of life in breast cancer survivors: A 10-year longitudinal cohort study. Quality of Life Research.

[B8-behavsci-15-01297] Chung J. O. K., Li W. H. C., Cheung A. T., Ho L. L. K., Xia W., Chan G. C. F., Lopez V. (2021). Relationships among resilience, depressive symptoms, self-esteem, and quality of life in children with cancer. Psycho-Oncology.

[B9-behavsci-15-01297] Clair M., Daniel C., Lamont M. (2016). Destigmatization and health: Cultural constructions and the long-term reduction of stigma. Social Science & Medicine.

[B10-behavsci-15-01297] Cohen S., Wills T. A. (1985). Stress, social support, and the buffering hypothesis. Psychological Bulletin.

[B11-behavsci-15-01297] Deci E. L., Ryan R. M. (2000). The “what” and “why” of goal pursuits: Human needs and the self-determination of behavior. Psychological Inquiry.

[B12-behavsci-15-01297] Dew M. A., Rosenberger E. M., Myaskovsky L., DiMartini A. F., Dabbs A. J. D., Posluszny D. M., Steel J., Switzer G. E., Shellmer D. A., Greenhouse J. B. (2016). Depression and anxiety as risk factors for morbidity and mortality after organ transplantation: A systematic review and meta-analysis. Transplantation.

[B13-behavsci-15-01297] Ding Y., Zhang H., Hu Z., Sun Y., Wang Y., Ding B., Yue G., He Y. (2024). Perceived social support and health-related quality of life among hypertensive patients: A latent profile analysis and the role of delay discounting and living alone. Risk Management and Healthcare Policy.

[B14-behavsci-15-01297] Dun L., Xian-Yi W., Si-Ting H. (2022). Effects of cognitive training and social support on cancer-related fatigue and quality of life in colorectal cancer survivors: A systematic review and meta-analysis. Integrative Cancer Therapies.

[B15-behavsci-15-01297] El Hadi S. N., Zanotti R., Danielis M. (2025). Lived experiences of persons with heart transplantation: A systematic literature review and meta-synthesis. Heart & Lung.

[B16-behavsci-15-01297] Fatma C., Cigdem C., Emine C., Omer B. (2021). Life experiences of adult heart transplant recipients: A new life, challenges, and coping. Quality of Life Research.

[B17-behavsci-15-01297] Fillingim M., Tanguay-Sabourin C., Parisien M., Zare A., Guglietti G. V., Norman J., Petre B., Bortsov A., Ware M., Perez J. (2025). Biological markers and psychosocial factors predict chronic pain conditions. Nature Human Behaviour.

[B18-behavsci-15-01297] Gao C., Gui S., Zhu L., Bian X., Shen H., Jiao C. (2025). Social support and quality of life in Chinese heart transplant recipients: Mediation through uncertainty in illness and moderation by psychological resilience. Frontiers in Psychiatry.

[B19-behavsci-15-01297] Goodwin R., Costa P., Adonu J. (2004). Social support and its consequences: ‘Positive’and ‘deficiency’values and their implications for support and self-esteem. British Journal of Social Psychology.

[B20-behavsci-15-01297] Greenberg J., Pyszczynski T., Solomon S. (1986). The causes and consequences of a need for self-esteem: A terror management theory. Public self and private self.

[B21-behavsci-15-01297] Grossman M. (2022). Supportive care and end of life. Promoting healing and resilience in people with cancer: A nursing perspective.

[B22-behavsci-15-01297] Guan L., Wu T., Yang J., Xie X., Han S., Zhao Y. (2020). Self-esteem and cultural worldview buffer mortality salience effects on responses to self-face: Distinct neural mediators. Biological Psychology.

[B23-behavsci-15-01297] Hajihasani M., Naderi N. (2021). Death anxiety in the elderly: The role of spiritual health and perceived social support. Aging Psychology.

[B24-behavsci-15-01297] Hashim M., Azim W., Hussain W., Rehman F. U., Salam A., Rafique M. (2022). Quality of life, perceived social support and death anxiety among people having cardiovascular disorders: A cross-sectional study. Pakistan Journal of Medical & Health Sciences.

[B25-behavsci-15-01297] Hayes A. F. (2017). Introduction to mediation, moderation, and conditional process analysis: A regression-based approach.

[B26-behavsci-15-01297] Hettwer M. D., Dorfschmidt L., Puhlmann L. M., Jacob L. M., Paquola C., Bethlehem R. A., Consortium N., Bullmore E. T., Eickhoff S. B., Valk S. L. (2024). Longitudinal variation in resilient psychosocial functioning is associated with ongoing cortical myelination and functional reorganization during adolescence. Nature Communications.

[B27-behavsci-15-01297] Hong Y., Yuhan L., Youhui G., Zhanying W., Shili Z., Xiaoting H., Wenhua Y. (2022). Death anxiety among advanced cancer patients: A cross-sectional survey. Supportive Care in Cancer.

[B28-behavsci-15-01297] Hu N., Yang Z., Wang A. (2024). Early post-transplant adaptation experience in young and middle-aged people with kidney transplant in China: A qualitative study. Western Journal of Nursing Research.

[B29-behavsci-15-01297] Huang Y., Guan Z., Yan F., Wiley J. A., Reynolds N. R., Tang S., Sun M. (2022). Mediator role of presence of meaning and self-esteem in the relationship of social support and death anxiety. Frontiers in Psychology.

[B30-behavsci-15-01297] IBM Corp (2023). IBM SPSS statistics for windows *(Version 27.0) [Computer software]*.

[B31-behavsci-15-01297] Ishikawa A., Rickwood D., Bariola E., Bhullar N. (2023). Autonomy versus support: Self-reliance and help-seeking for mental health problems in young people. Social Psychiatry and Psychiatric Epidemiology.

[B32-behavsci-15-01297] Iverach L., Menzies R. G., Menzies R. E. (2014). Death anxiety and its role in psychopathology: Reviewing the status of a transdiagnostic construct. Clinical Psychology Review.

[B33-behavsci-15-01297] Jaberi M., Mohammadi T. K., Adib M., Maroufizadeh S., Ashrafi S. (2025). The relationship of death anxiety with quality of life and social support in hemodialysis patients. OMEGA-Journal of Death and Dying.

[B34-behavsci-15-01297] Ji P., Zhang L., Gao Z., Ji Q., Xu J., Chen Y., Song M., Guo L. (2024). Relationship between self-esteem and quality of life in middle-aged and older patients with chronic diseases: Mediating effects of death anxiety. BMC Psychiatry.

[B35-behavsci-15-01297] Ji Q., Zhang L., Xu J., Ji P., Song M., Chen Y., Guo L. (2024). The relationship between stigma and quality of life in hospitalized middle-aged and elderly patients with chronic diseases: The mediating role of depression and the moderating role of psychological resilience. Frontiers in Psychiatry.

[B36-behavsci-15-01297] Jiang N., Ye H., Zhao X., Zhang Y. (2025). The association between social support and the quality of life of older adults in China: The mediating effect of loneliness. Experimental Aging Research.

[B37-behavsci-15-01297] Kandeğer A., Aydın M., Altınbaş K., Cansız A., Tan Ö., Tomar Bozkurt H., Eğilmez Ü., Tekdemir R., Şen B., Aktuğ Demir N. (2021). Evaluation of the relationship between perceived social support, coping strategies, anxiety, and depression symptoms among hospitalized COVID-19 patients. The International Journal of Psychiatry in Medicine.

[B38-behavsci-15-01297] Khanbabai Ghaleie P., Hooman F., Naderi F., Talebzadeh Shoushtari M., Gatezadeh A. (2024). The role of social support and spiritual health in predicting death anxiety in patients with cancer. Hospital Practices and Research.

[B39-behavsci-15-01297] Kisomi Z. S., Taherkhani O., Mollaei M., Esmaeily H., Shirkhanloo G., Hosseinkhani Z., Amerzadeh M. (2024). The moderating role of social support in the relationship between death anxiety and resilience among dialysis patients. BMC Nephrology.

[B40-behavsci-15-01297] Knobbe T. J., Kremer D., Bültmann U., Annema C., Navis G., Berger S. P., Bakker S. J. L., Meuleman Y. (2025). Insights into health-related quality of life of kidney transplant recipients: A narrative review of associated factors. Kidney Medicine.

[B41-behavsci-15-01297] Kristianto Y. D., Yudiarso A. (2025). A meta-analysis correlation social support and quality of life. Jurnal Psikologi Tabularasa.

[B42-behavsci-15-01297] Kuo B. C. (2013). Collectivism and coping: Current theories, evidence, and measurements of collective coping. International Journal of Psychology.

[B43-behavsci-15-01297] Lazarus R. S., Folkman S. (1984). Stress, appraisal, and coping.

[B44-behavsci-15-01297] Lehto R., Stein K. F. (2009). Death anxiety: An analysis of an evolving concept. Research and Theory for Nursing Practice: An International Journal.

[B45-behavsci-15-01297] Levine G. N., Cohen B. E., Commodore-Mensah Y., Fleury J., Huffman J. C., Khalid U., Labarthe D. R., Lavretsky H., Michos E. D., Spatz E. S. (2021). Psychological health, well-being, and the mind-heart-body connection: A scientific statement from the American Heart Association. Circulation.

[B46-behavsci-15-01297] Li G., Li Y., Lam A. I. F., Tang W., Seedat S., Barbui C., Papola D., Panter-Brick C., Waerden J. V., Bryant R., Mittendorfer-Rutz E., Gémes K., Purba F. D., Setyowibowo H., Pinucci I., Palantza C., Acarturk C., Kurt G., Tarsitani L., Hall B. J. (2023). Understanding the protective effect of social support on depression symptomatology from a longitudinal network perspective. BMJ Ment Health.

[B47-behavsci-15-01297] Li Z., Qin S., Zhu Y., Zhou Q., Yi A., Mo C., Gao J., Chen J., Wang T., Feng Z. (2025). Social support mediates the relationship between depression and subjective well-being in elderly patients with chronic diseases: Evidence from a survey in Rural Western China. PLoS ONE.

[B48-behavsci-15-01297] Lin C., Zhu X., Wang X., Wang L., Wu Y., Hu X., Wen J., Cong L. (2025). The impact of perceived social support on chronic disease self-management among older inpatients in China: The chain-mediating roles of psychological resilience and health empowerment. BMC Geriatrics.

[B49-behavsci-15-01297] Lin D., Liang D., Huang M., Xu X., Bai Y., Meng D. (2024). The dyadic effects of family resilience and social support on quality of life among older adults with chronic illness and their primary caregivers in multigenerational families in China: A cross-sectional study. Heliyon.

[B50-behavsci-15-01297] Lin S., Liu D., Niu G., Longobardi C. (2022). Active social network sites use and loneliness: The mediating role of social support and self-esteem. Current Psychology.

[B51-behavsci-15-01297] Liu J., Chang S., Wang Z., Raja F. Z. (2024). Exploring the association between social support and anxiety during major public emergencies: A meta-analysis of the COVID-19 pandemic. Front Public Health.

[B52-behavsci-15-01297] Liu X., Bai S. (2024). Downward intergenerational support and well-being in older chinese adults. International Journal of Environmental Research and Public Health.

[B53-behavsci-15-01297] Luo R., Ji Y., Liu Y. h., Sun H., Tang S., Li X. (2023). Relationships among social support, coping style, self-stigma, and quality of life in patients with diabetic foot ulcer: A multicentre, cross-sectional study. International Wound Journal.

[B54-behavsci-15-01297] Mauthner O. E., De Luca E., Poole J. M., Abbey S. E., Shildrick M., Gewarges M., Ross H. J. (2015). Heart transplants: Identity disruption, bodily integrity and interconnectedness. Health.

[B55-behavsci-15-01297] Merati M., Jalali A., Naghibzadeh A., Salari N., Moradi K. (2024). Study of the relationship between death anxiety and quality of life in patients with heart failure. OMEGA-Journal of Death and Dying.

[B56-behavsci-15-01297] Milaniak I., Rużyczka E. W., Dębska G., Król B., Wierzbicki K., Tomaszek L., Przybyłowski P. (2020). Level of life quality in heart and kidney transplant recipients: A multicenter study. Transplantation Proceedings.

[B57-behavsci-15-01297] Papalois Z.-A., Papalois V. (2023). Health-related quality of life and patient reported outcome measures following transplantation surgery. Patient reported outcomes and quality of life in surgery.

[B58-behavsci-15-01297] Podsakoff P. M., MacKenzie S. B., Lee J.-Y., Podsakoff N. P. (2003). Common method biases in behavioral research: A critical review of the literature and recommended remedies. Journal of Applied Psychology.

[B59-behavsci-15-01297] Qin S., Sheehan L., Yau E., Chen Y., Wang Y., Deng H., Lam C., Chen Z., Zhao L., Corrigan P., CBPR Team (2024). Adapting and evaluating a strategic disclosure program to address mental health stigma among Chinese. International Journal of Mental Health and Addiction.

[B60-behavsci-15-01297] Reed B., Rea K. E., Claar R. L., van Tilburg M. A., Levy R. L. (2021). Passive coping associations with self-esteem and health-related quality of life in youth with inflammatory bowel disease. Frontiers in Psychology.

[B61-behavsci-15-01297] Religioni U., Barrios-Rodríguez R., Requena P., Borowska M., Ostrowski J. (2025). Enhancing Therapy adherence: Impact on clinical outcomes, healthcare costs, and patient quality of life. Medicina.

[B62-behavsci-15-01297] Rimé B., Bouchat P., Paquot L., Giglio L. (2020). Intrapersonal, interpersonal, and social outcomes of the social sharing of emotion. Current Opinion in Psychology.

[B63-behavsci-15-01297] Rosenberg M. (1965). Rosenberg self-esteem scale. Journal of Religion and Health.

[B64-behavsci-15-01297] Rosenberger E. M., Fox K. R., DiMartini A. F., Dew M. A. (2012). Psychosocial factors and quality-of-life after heart transplantation and mechanical circulatory support. Current Opinion in Organ Transplantation.

[B65-behavsci-15-01297] Ross H., Abbey S., De Luca E., Mauthner O., McKeever P., Shildrick M., Poole J. (2010). What they say versus what we see:“Hidden” distress and impaired quality of life in heart transplant recipients. The Journal of Heart and Lung Transplantation.

[B66-behavsci-15-01297] Sadjapong U., Thongtip S. (2023). Association between self-esteem and health-related quality of life among elderly rural community, Northern Thailand. Cogent Social Sciences.

[B67-behavsci-15-01297] Saltzman L. Y., Hansel T. C. (2024). Psychological and social determinants of adaptation: The impact of finances, loneliness, information access and chronic stress on resilience activation. Frontiers in Psychology.

[B68-behavsci-15-01297] Schabert J., Browne J. L., Mosely K., Speight J. (2013). Social stigma in diabetes: A framework to understand a growing problem for an increasing epidemic. The Patient-Patient-Centered Outcomes Research.

[B69-behavsci-15-01297] Shildrick M. (2015). Staying alive: Affect, identity and anxiety in organ transplantation. Body & Society.

[B70-behavsci-15-01297] Smirnov E., Makarova M. (2025). Existential concerns arising from a threat to the belief in a just world: A mixed-methods study. Journal of Humanistic Psychology.

[B71-behavsci-15-01297] Smith M. L., Bergeron C. D., Riggle S. D., Meng L., Towne S. D., Ahn S., Ory M. G. (2017). Self-care difficulties and reliance on support among vulnerable middle-aged and older adults with chronic conditions: A cross-sectional study. Maturitas.

[B72-behavsci-15-01297] Sun Z., Zhao L., Wei H., Wang X., Riemersma R. R. (2023). Do guanxi and harmonious leadership matter in the sociocultural integration by Chinese multinational enterprises in The Netherlands?. International Journal of Emerging Markets.

[B73-behavsci-15-01297] Surzykiewicz J., Skalski S. B., Sołbut A., Rutkowski S., Konaszewski K. (2022). Resilience and regulation of emotions in adolescents: Serial mediation analysis through self-esteem and the perceived social support. International Journal of Environmental Research and Public Health.

[B74-behavsci-15-01297] Svenaeus F. (2012). Organ transplantation and personal identity: How does loss and change of organs affect the self?. Journal of Medicine and Philosophy.

[B75-behavsci-15-01297] Svet M., Portalupi L. B., Pyszczynski T., Matlock D. D., Allen L. A. (2023). Applying terror management theory to patients with life-threatening illness: A systematic review. BMC Palliative Care.

[B76-behavsci-15-01297] The WHOQOL Group (1995). The World Health Organization quality of life assessment (WHOQOL): Position paper from the World Health Organization. Social Science & Medicine.

[B77-behavsci-15-01297] Thompson D. M., Booth L., Moore D., Mathers J. (2022). Peer support for people with chronic conditions: A systematic review of reviews. BMC Health Services Research.

[B78-behavsci-15-01297] Vail K. E., Reed D. E., Goncy E. A., Cornelius T., Edmondson D. (2020). Anxiety buffer disruption: Self-evaluation, death anxiety, and stressor appraisals among low and high posttraumatic stress symptom samples. Journal of Social and Clinical Psychology.

[B79-behavsci-15-01297] van Schie C. C., Chiu C. D., Rombouts S., Heiser W. J., Elzinga B. M. (2018). When compliments do not hit but critiques do: An fMRI study into self-esteem and self-knowledge in processing social feedback. Social Cognitive and Affective Neuroscience.

[B80-behavsci-15-01297] Wang L.-S., Yen P.-T., Weng S.-F., Hsu J.-H., Yeh J.-L. (2022). Clinical patterns of traditional Chinese medicine for ischemic heart disease treatment: A population-based cohort study. Medicina.

[B81-behavsci-15-01297] Wang P., Gao H., Xu J., Huang J., Wang C. (1998). Reliability and validity study of self-esteem scale. Shandong Psychiatry.

[B82-behavsci-15-01297] Wang Y., Li F., Qiao W., Zhang J., Dong N. (2025). Global challenges and development of heart transplantation: Wuhan Protocol development and clinical implementation. Chinese Medical Journal.

[B83-behavsci-15-01297] Ware J. E., Snow K. K., Kosinski M., Gandek B. (1993). SF-36 health survey: Manual and interpretation guide, 2.

[B84-behavsci-15-01297] White-Williams C., Grady K. L., Fazeli P., Myers S., Moneyham L., Meneses K., Rybarczyk B. (2014). The partial mediation effect of satisfaction with social support and coping effectiveness on health-related quality of life and perceived stress long-term after heart transplantation. Nursing: Research and Reviews.

[B85-behavsci-15-01297] Wu S., Liu H., Li Y., Teng Y. (2024). The influence of self-esteem on sociocultural adaptation of college students of Hong Kong, Macao and Taiwan: The chain mediating role of social support and school belonging. Psychology Research and Behavior Management.

[B86-behavsci-15-01297] Xiao S. (1994). Theoretical foundation and research applications of the social support rating scale. Journal of Clinical Psychiatry.

[B87-behavsci-15-01297] Xiong H.-X., Zhang J., Ye B.-J., Zheng X., Sun P.-Z. (2012). Common method variance effects and the models of statistical approaches for controlling it. Advances in Psychological Science.

[B88-behavsci-15-01297] Yan J., Tian J., Yang H., Han G., Liu Y., He H., Han Q., Zhang Y. (2022). The causal effects of anxiety-mediated social support on death in patients with chronic heart failure: A multicenter cohort study. Psychology Research and Behavior Management.

[B89-behavsci-15-01297] Yang H., Li Y., Yao Q., Wen X. (2013). The application of the Chinese version of the death anxiety scale and its implications for death education. Journal of Nursing.

[B90-behavsci-15-01297] Zambrano J., Celano C. M., Januzzi J. L., Massey C. N., Chung W. J., Millstein R. A., Huffman J. C. (2020). Psychiatric and psychological interventions for depression in patients with heart disease: A scoping review. Journal of the American Heart Association.

[B91-behavsci-15-01297] Zech E., Rimé B., Nils F. (2004). Social sharing of emotion, emotional recovery, and interpersonal aspects. The regulation of emotion.

[B92-behavsci-15-01297] Zhang D. (2021). On the paradox of Wuwei—A refutation and defense of Daoist “right action”. Philosophical Trends.

[B93-behavsci-15-01297] Zhang D., Zhang N., Chang H., Shi Y., Tao Z., Zhang X., Miao Q., Li X. (2023). Mediating role of hope between social support and self-management among Chinese liver transplant recipients: A multi-center cross-sectional study. Clinical Nursing Research.

[B94-behavsci-15-01297] Zhang Z., Jin J., Luo C., Chen A. (2022). Excavating the social representations and perceived barriers of organ donation in China over the past decade: A hybrid text analysis approach. Front Public Health.

[B95-behavsci-15-01297] Zheng Z. (2025). Exploration and prospects for the high quality development of standardized heart transplantation in China. Chinese Circulation Journal.

[B96-behavsci-15-01297] Zhou S., Liu G., Huang Y., Huang T., Lin S., Lan J., Yang H., Lin R. (2023). The contribution of cultural identity to subjective well-being in collectivist countries: A study in the context of contemporary Chinese culture. Frontiers in Psychology.

